# Training systems affect spatial distribution of Korla fragrant pear (*Pyrus sinkiangensis* Yu) fruits by altering canopy structure and light distribution

**DOI:** 10.3389/fpls.2025.1615019

**Published:** 2025-07-29

**Authors:** Pan Yan, Yonghui Deng, Shi-jie An, Ling Ma, Tianle Li, Qi-ling Chen, Qiangqing Zheng

**Affiliations:** Institute of Forestry and Horticulture of Xinjiang Academy of Agricultural and Reclamation Science, Tiemenguan Test Station of Xinjiang Academy of Agricultural and Reclamation Science, Xinjiang Production & Construction Corp Key Laboratory of Korla Fragrant Pear Germplasm Innovation and Quality Improvement and Efficiency Increment, Shihezi, China

**Keywords:** Pyrus sinkiangensis Yu, canopy structure, light distribution, fruit spatial distribution, yield

## Abstract

**Objective:**

This study aims to elucidate the relationship between canopy structure and fruit spatial distribution, establish a model linking canopy light distribution with fruit positioning, and identify optimal training strategies for consistently high yield. The findings provide a theoretical foundation for optimizing modern cultivation practices in Korla fragrant pear orchards.

**Methods:**

Four training treatments were established: precision pruning, reduction, falling head, and thinning. Canopy structural parameters and light distribution were measured, along with canopy light interception (ALI). Fruit number and individual fruit weight were recorded at different canopy positions. Correlation analysis was used to revealing the relationship between canopy structure, light distribution, and spatial distribution of fruits.

**Results:**

(1) Canopy Structure: Reduction and falling head effectively controlled canopy spread, significantly reduced the proportion of long branches while increased the proportion of middle branches. Thinning, however, increased the proportion of long branches, total branch length and average branch length, led to significant expansion in canopy diameter, surface area, and volume. (2) Light Distribution: Reduction increased average light interception (ALI) by 15%, while thinning improved ALI by 11% significantly, enhanced light availability across different canopy aspects, falling head notably improved light penetration in the middle and lower canopy layers. Persistent low-light zones (ALI < 300 μmol·m^-^²·s^-^¹) were identified in the lower canopy, inner canopy, and inter-tree spaces, highlighted key areas for light optimization. (3) Fruit Spatial Distribution: Smaller canopies had fewer but more uniformly distributed fruits. As canopy size increased, light interception and photosynthesis improved, total fruit yield improved, however, spatial heterogeneity intensified, with fruit-bearing zones shifted outward and upward, reduced carbon allocation uniformity. (4) Yield Correlations: Two canopy structural parameters showed significant negative correlations with consistently high yield traits, while ten exhibited positive correlations. Tree height, canopy surface area, and total branch length had the strongest positive associations with total yield. (5) Key Relationship: The correlation between light distribution and fruit spatial distribution strengthened significantly with canopy expansion.

**Conclusion:**

The influence of canopy structure and light distribution on fruit spatial distributions depends on canopy size. For small canopies, canopy structure serves as the dominant factor affecting fruit distribution, while in large canopies, light distribution becomes the primary driver. Accordingly, distinct canopy management strategies should be adopted, small canopies should focus on increasing canopy surface area and total branch length, to increase fruiting sites and enhance yield, large canopies require optimizing light distribution to improve fruit spatial uniformity. Thinning promoted flower bud formation significantly by increased the proportion of long branches and lateral branch number, thereby optimized consistently high yield traits.

## 1 Introduction

### 1.1 Research significance

Understanding the structure, light distribution, and fruit spatial distribution of fruit trees at the canopy scale is crucial for optimizing orchard system design, maximizing the utilization of light resources, and unlocking production potential (Wertheim et al., 2001; Tustin et al., 2022). Canopy structure is a key factor influencing fruit spatial distribution, as its shoot and leaf traits and arrangement determine the quantity and positioning of flower buds. Light distribution is another critical factor potentially affecting fruit spatial distribution, given the close relationship between flower bud differentiation processes and light conditions (Wagenmakers and Callesen, 2015). However, accurately quantifying canopy structure, light distribution, and fruit spatial distribution in fruit trees – and elucidating their complex interrelationships – remains challenging due to the large size and intricate architecture of tree crowns (Díaz Espejo et al., 2008; Zhu et al., 2020; Murcia et al., 2022). To address this challenge, we conducted a three-year study, established a comprehensive evaluation index system for the canopy structure of Korla fragrant pear (*Pyrus sinkiangensis* Yu), innovated methods for measuring canopy light distribution and evaluating light interception, and combined these with fruit mapping techniques, to elucidate the quantitative relationship between canopy structure and fruit spatial distribution, establish a predictive model correlating light distribution with fruit positioning patterns, and develop optimization strategies for cultivation systems to enhance both light use efficiency and yield productivity.

### 1.2 Research progress

The relationship between canopy structure and fruit spatial distribution. At the canopy scale, research has explored tree architecture in labor-efficient pear orchards and its relationship with yield characteristics (Yang, 2013; Ding, 2021; Li et al., 2024). Studies on 12-year-old Korla fragrant pear trees trained with an open-center system found the primary fruiting zone concentrated in the middle and upper canopy layers, within 1.5 m of the trunk (Zari et al., 2016). For 11-year-old pear trees with a small, sparse-layered canopy, the highest-yielding zone (accounting for 77% of total yield) was located between 1.0 m and 2.0 m in height. Similarly, in 6-year-old trellis-trained ‘Whangkeumbae’ pears, the peak yield zone occurred between 1.0 and 1.5 m height. Horizontally, the high-yield areas for both training systems were centered within the canopy (Li et al., 2009; Yue et al., 2008). These findings collectively demonstrate that distinct canopy structures result in different spatial concentrations of fruit, creating unique spatial distribution patterns primarily determined by shoot traits and arrangement. At the shoot scale, shoots serve as the fundamental units bearing fruit and dictate flower and fruit formation. Shoot growth, flowering intensity, fruit set, and fruit size collectively influence canopy zone productivity (Acebedo et al., 2000). Shoots exhibit a degree of autonomy in carbon economy, exerting localized control over carbon uptake and partitioning. Manipulating the distribution of shoots, leaves, and fruits through pruning alters source-sink relationships and impacts carbon allocation (Marsal et al., 2003; Lang et al., 2004). Furthermore, shoot type and leaf-to-fruit distance also influence carbon partitioning (Sha et al., 2020). Significant differences in fruit number have been observed among different scaffold branches (Li et al., 2018), with fruit clustering occurring even within individual branches (Zari et al., 2016). Shoot (and foliage) quantity and basal diameter show significant positive correlations with individual tree yield (Wu, 2023), with correlation coefficients reaching up to 0.70 (Lu et al., 2011). Well-developed fruiting units with a higher proportion of medium and long shoots promote robust canopy structure, renewal, and rejuvenation, providing a foundation for high and consistent yields (Liu et al., 2023). Long, vigorous shoots exhibit significantly higher yields than short, thin shoots (Gao, 2020). These studies underscore the impact of shoot traits on fruit quantity. While the influence of shoot distribution and morphology on fruit distribution is evident, its comprehensive quantification, particularly at the whole-tree level, remains incomplete (Wang et al., 2023). Therefore, this study established a novel evaluation index system for the canopy structure of Korla fragrant pear (*Pyrus sinkiangensis* Yu). This system incorporates canopy-scale parameters (e.g., height, spread) alongside shoot-scale parameters (e.g., shoot length, shoot type composition). Fruit distribution was precisely mapped using spatial positioning techniques. This integrated approach is designed to more effectively elucidate the mechanisms by which canopy structure influences fruit spatial heterogeneity.

The relationship between light distribution and fruit spatial distribution. Research has demonstrated that plants grown under high light availability exhibit higher reproductive success than those under low light conditions (Svriz et al., 2023). Within tree canopies, the uneven distribution of light—where some branches receive ample sunlight while others remain shaded—leads to heterogeneous carbon budgets across the canopy, consequently influencing fruit distribution (Marsal et al., 2003). When tree height exceeds 4 m and the leaf area index (LAI) surpasses 2.0, light intensity in the lower and inner canopy typically falls below 30% of ambient light. Consequently, leaves in these regions exhibit lower net photosynthetic rate (Pn), specific leaf weight, chlorophyll content, and smaller flower bud size compared to outer canopy leaves. Therefore, excessive tree height should be avoided to improve light penetration (Han et al., 2001; Yoon and Bhusal, 2016; Bhusal et al., 2019). Canopy thinning via pruning can also enhance internal light levels, thereby increasing the number of flowering sites and improving fruit set in crops like olive (Trentacoste et al., 2018; Rallo et al., 2024). In raspberry, both fruit number per unit leaf area and per lateral node increase with higher light exposure. Fruit set reached approximately 90% on laterals exposed to afternoon full-sun photosynthetic active radiation (FS-PAR) greater than 25%, compared to only about 46% for laterals exposed to 0–25% FS-PAR (Braun et al., 2015). It is noteworthy that canopy structure directly influences light distribution within the canopy (Béland and Baldocchi, 2021). As canopy size increases, shaded areas frequently develop inside the crown (Wagenmakers and Callesen, 2015), led to significant variation in photosynthetically active radiation (PAR) among different canopy positions - with differences reaching up to 10-fold (Araujo et al., 2008; Song et al., 2014). Consequently, canopy structure and light distribution represent intrinsically linked independent variables, the influence on fruit spatial distribution is multifaceted in nature.

Current research limitations and contributions of this study. Currently, the relationship between canopy structure and fruit spatial distribution in fruit trees remains unclear, and whether light distribution plays a key role in fruit positioning is still uncertain. This knowledge gap has constrained the optimization of cultivation systems and yield improvement. The primary limitations include: (1) the lack of quantitative evaluation metrics for canopy structure, and (2) insufficient detailed surveys of fruit spatial distribution, which collectively hinder a deeper understanding of canopy characteristics and their relationship with fruit positioning patterns. This study makes significant contributions by: establishing a novel evaluation index system for Korla fragrant pear canopy structure that incorporates: canopy-scale parameters (height, canopy diameter), branch-scale parameters (branch length, branch-type composition), thereby enhancing the comprehensiveness of structural quantification. Regarding canopy light distribution, conventional studies have predominantly relied on grid-based methods, which suffer from low measurement efficiency and fail to provide a comprehensive assessment of the canopy light environment. This study developing an innovative 3D quadrant method for light distribution analysis that: optimizes sensor placement to better represent actual canopy geometry, increases measurement density, incorporates temporal dynamics, enabling spatiotemporal evaluation of light distribution and quantitative assessment of light interception. At last, implementing precise 3D fruit positioning through the quadrant method, enabling accurate spatial distribution mapping position-dependent yield analysis. These methodological advances provide critical tools for precisely regulating canopy structure and light environment, with important implications for improving light use efficiency and yield potential in orchard systems.

### 1.3 Research focus

This study designed distinct training treatments to shape varied canopy structures, enabling quantitative analysis of canopy structure and light distribution, alongside precise spatial mapping of fruit positioning patterns.

### 1.4 Key objectives

Elucidate the relationship between canopy structure and fruit spatial distribution. Establish a predictive model correlating photosynthetically active radiation (PAR) intensity with fruit set quantity. The findings will provide a theoretical framework for: optimizing canopy structural parameters, improving light interception efficiency, enhancing carbon assimilation and allocation dynamics, maximizing yield potential.

## 2 Materials and methods

### 2.1 Overview of the experimental site

The experiment was conducted from 2022 to 2024 in a labor-saving and densely planted Korla fragrant pear orchard at Regiment 29, Division 2 of the Xinjiang Production and Construction Corps. The experimental site is located in the northeastern Tarim Basin (41.8°N, 85.7°E), with an annual sunshine duration of 2,990 h, a frost-free period of 210 d, and a mean annual temperature of 11.4°C. Recent two-year averages included a high temperature of 17°C (extreme high: 35°C) and a low temperature of 6°C (extreme low: -23°C). The mean annual precipitation was 58.6 mm, and the maximum annual evaporation reached 2,788.2 mm. The precision pruning orchard was planted in 2016 and grafted in 2017, while the other treated orchard was planted in 2014 and grafted in 2015. The soil is sandy loam with a pH of 7.99, total salt content of 2,022 μS·cm^-^¹, organic matter of 6.5 g·kg^-^¹, total nitrogen of 0.4 g·kg^-^¹, available phosphorus of 7.6 mg·kg^-^¹, and available potassium of 323 mg·kg^-^¹. Border irrigation and conventional fertilization were applied. Rootstocks were all Pyrus betulifolia (birchleaf pear), with rows oriented east-west.

### 2.2 Experimental methods

**Figure 1:**
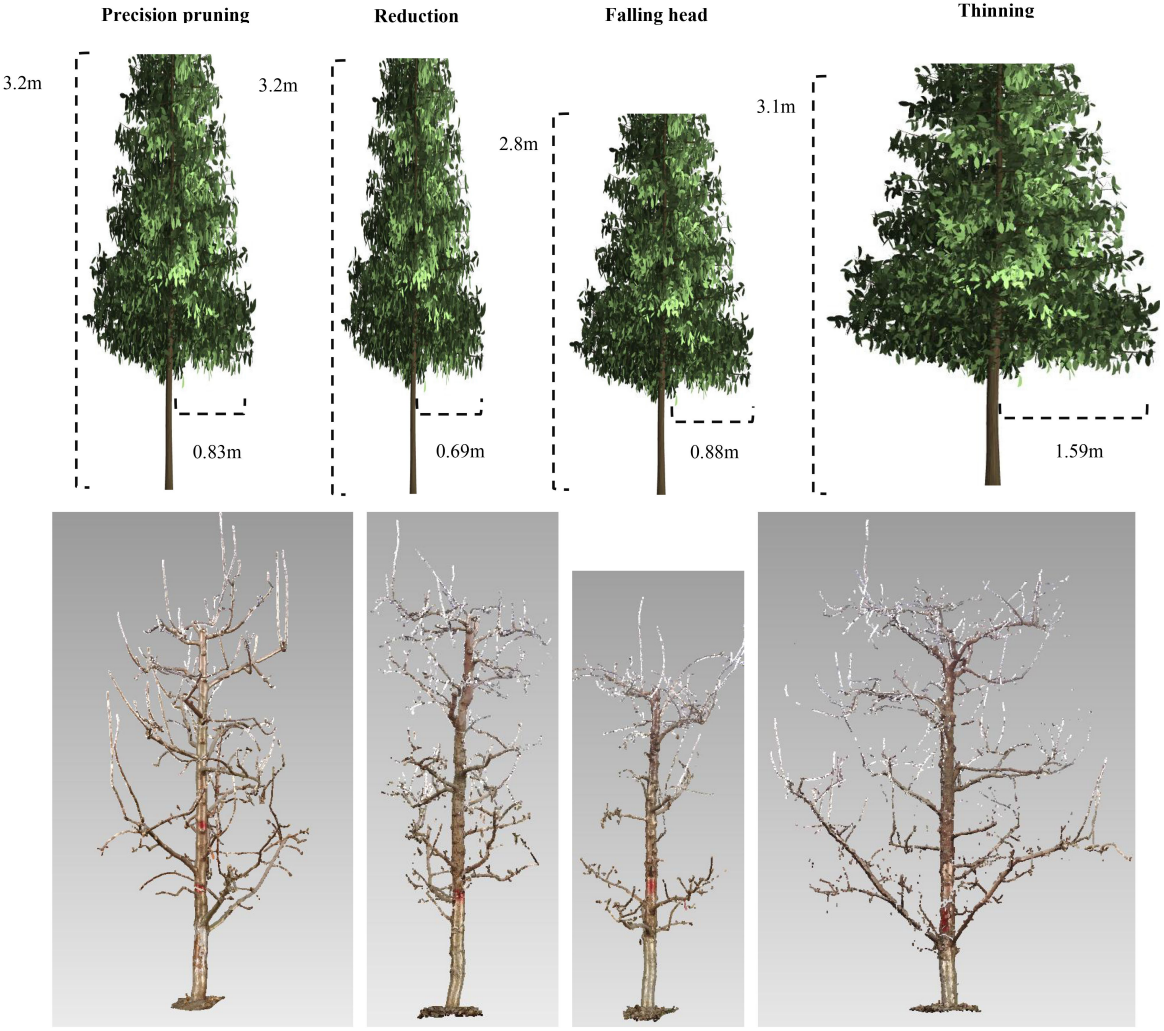
Training systems and canopy structure.

The experiment followed a single-factor design, four training systems were applied to train tree canopies into specific shapes:(1)Precision Pruning (PP) – Primarily regulates branch composition to balance the proportion of long, middle, and short branches. (2)Reduction (RD) – Targets long shoots to promote middle branch dominance. (3) Falling Head (FH) – Controls tree height by lowering the canopy. (4) Thinning (TN) – Adjusts spacing between trees to expand growing space. The resulting canopy shapes included: Slender Spindle (SS), Narrow Cylindrical (NC), Short Cylindrical (SC), Wide Cylindrical (WC), scan the canopy structure with a laser scanner FARO S70 in Winter 2024 (**Figure 1**). The purpose of all treatments is to improve canopy light distribution, enhance yield and quality. Each treatment comprised 100 trees with a spacing of 1 m×4 m. For RD and FH treatments, pruning was conducted during summer 2022. TN involved no summer pruning in 2022 but was thinning in winter 2022, to ensure growth space, adjusting the spacing to 2 m×4 m post-thinning. In summer 2023, after pruning, three representative trees per treatment were selected to assess tree structure, canopy light distribution and fruit number at different canopy parts for high yield traits analysis. In 2024, fruit set and flower bud number of these trees were further evaluated to analyze stable yield traits.

Determination of canopy structure parameters: including ground diameter, tree height, branch number, branch length (excluding new shoots of the year), branch diameter, branch angle, distance of branches, lateral branch number, etc. The ground diameter was measured at 30 cm from the ground using a breast diameter scale. The tree height is measured by tower ruler, close to the central trunk, with the ground as the lowest point and the top of the central trunk as the highest point. The number of branches is counted from bottom to top. The branch length is measured from the base of the branch to the top of the branch, excluding the new shoots issued in the year. Branch diameter At the branch of the central stem, the diameter of the base of the branch was measured with a vernier caliper. The branch angle is measured in the middle of the branch with a digital angle ruler, which represents the angle of the longest section of the branch. The lower side of the angle ruler is horizontal, and the upper side is parallel to the branch. The distance of branches was measured with a ruler. Long, medium and short branches, lower, middle and upper layers were counted. The branch length smaller than 50 cm was classified as short branches, between 50 cm and 100 cm was classified as middle branch, larger 100 cm was classified as long branch. The number of lateral branches included short lateral branches with length > 2 cm on the main branch. Canopy diameter: Ave(Average branch length per layer×cos(90-A)×2)/100, A:Branch angle, the angle between the branch and the central trunk, 100: Conversion of units, cm-m. Canopy surface area: Sum(Side area of each cylinder layer)+Top circular area of upper layer, Side area of each layer=3.14×Canopy diameter×Height of each layer, Top circular area of upper layer=3.14×(Canopy diameter of upper layer/2)^2^. Canopy volume: Sum(Volume of each cylinder layer), volume of each cylinder layer=3.14*(Canopy diameter of upper layer/2)^2^×Height of each layer.

### 2.3 Determination of canopy light distribution and photosynthetic rate

**Figure 2:**
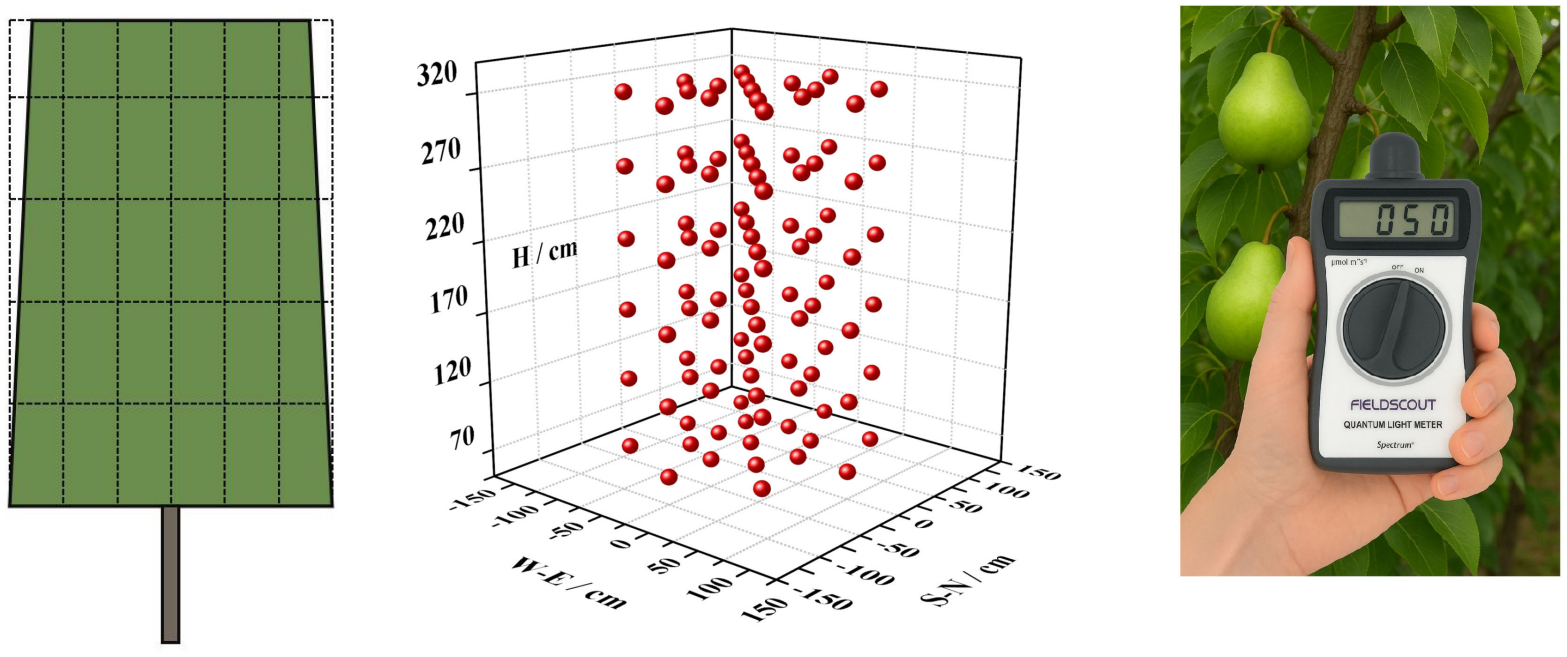
Schematic diagram of canopy light distribution measurement.

In June 2023, in order to avoid the influence of inter-annual weather, before and after the summer solstice when the solar elevation angle was the largest, continuous sunny days were selected for measurement. Three standard trees were selected for each tree shape, and the z-axis perpendicular to the quadrant was added on the basis of the quadrant (plane rectangular coordinate system) to construct a three-dimensional coordinate system. Taking the trunk as the z-axis, the row direction as the x-axis, and the straight line perpendicular to the row direction as the y-axis, the plane is divided into 8 quadrants, each quadrant is 45°, and the z-axis is divided into 6 layers: 70, 120, 170, 220, 270, 320 cm. The measurement points were distributed on the coordinate axis and diagonal of each layer quadrant, and were measured at 0, 50 and 100 cm according to the distance from the trunk. The east and west sides were measured at 0, 25 and 50 cm according to the plant distance, and the distance was shortened according to the plant distance, but the mapping was performed according to the 50 cm spacing (**Figure 2**). The PAR of different parts of the whole plant was measured every 2 hours from 10:00 - 20:00, the instrument was Spectrum 3415F photosynthetically active radiation measuring instrument, and the sensor was kept in a horizontal state during measurement. Light interception index system: (1) Light interception (LI): the PAR value received by a leaf at a certain time point in a certain part of the canopy, that is, the measured value of a sample point at a time point. (2) Average light interception (ALI): the average value of PAR received by leaves in different parts or periods of different tree shapes and canopy. ALI of different tree shapes: 6 periods per day, 6 layers per period, 8 orientations per layer, 3 distances from the trunk in each orientation, a total of 864 mean values of light interception (LI). ALI at different heights: 864 data per day, divided into 6 heights (layer spacing of 50 cm), the average of 144 LI per height. ALI at different distances from the trunk: 864 data per day, divided into 3 distances (50 cm per segment), and the average value of 288 LI per distance. ALI in different directions: 864 data in a single day, divided into 8 directions, and the average value of 108 LI in each direction. ALI at different times: 864 data of a single day, divided into 6 periods, the average value of 144 LI in each period. When making the light distribution map, the LI of each spatial point is represented by ALI at different times. Measure photosynthetic rate with the Li6400 XT photosynthesis system.

### 2.4 Determination of fruit distribution

On September 9th, different tree-shaped pear trees and pears in different canopy parts were collected. The sampling points corresponded to the light distribution measuring points. The fruit at 70 cm height was the fruit below 70 cm height, and the fruit at 120 cm height was in the 70 cm-120 cm area, and so on. Fruits in different orientations were defined as those located within a 45°sector centered on each cardinal direction (± 22.5°from the central axis). Fruits at different distances from the trunk were categorized into three segments: Fruits within 0–25 cm (marked at 0 cm position), Fruits within 25–75 cm (marked at 50 cm position), Fruits beyond 75 cm (marked at 100 cm position). The number of fruits in different parts was counted after harvest. The continuous fruiting ability was evaluated by the number of fruits and flower buds in the second year. The average number of fruit, the average number of flower buds per branch average.

### 2.5 Analysis methods

Photoshop was used to draw the schematic diagram. Excel 2016 software was used to sort out the data and draw the histogram. SPSS 21.0 software was used to analyze the variance and Duncan multiple comparison (α= 0.05). Oringin 9.1 was used to draw the scatter plot.

## 3 Results

### 3.1 The influence of different training systems on the canopy structure of Korla fragrant pear

The canopy structure exhibited significant variations among different training systems. Compared to precision pruning (PP), canopy width was reduced by reduction (RD), tree height was decreased by falling head (FH), and canopy expansion was promoted by thinning (TN). The specific parameters were as follows: RD significantly decreased the proportion of long branches by 60%, doubled the proportion of medium branches, increased average branch diameter by 20%, enlarged branch angles by 20%, and enhanced middle-layer branch diameter by 20% (p < 0.05), other parameters showed no significant differences. Falling head (FH) reduced tree height, compared to PP, FH significantly decreased tree height by 10%, reduced branch number by 30%, increased the proportion of middle branches by 90%, enlarged average branch diameter by 30%, expanded branch angles by 30%, decreased upper-layer branch number by 60%, and increased lower-layer and middle-layer branch diameter by 40% (p < 0.05), other parameters showed no significant differences. Thinning (TN) significantly increased the available growing space for tree canopys, led to a substantial increase in multiple canopy parameters, among these parameters, the trunk diameter increased by 20%, canopy diameter increased by 110%, canopy surface area increased by 170%, canopy volume increased by 400%, long branch proportion increased by 100%, total branch length increased by 60%, average branch length increased by 100%, branch diameter increased by 30%, total lateral branch number increased by 50%, and average lateral branch number increased by 100%. Branch length, diameter, and number in all canopy layers also increased significantly, except for a 40% reduction in upper-layer branch number (p < 0.05) (**Tables 1**–**3**).

**Table 1:** Canopy structure parameters of Korla fragrant pear under different training systems.

Training systems	Diameter(cm)	Height(m)	Canopy diameter(m)	Canopy surface area (m^2^)	Canopy volume(m^3^)	Branch number	Proportion of long branches (%)	Proportion of middle branches (%)	Proportion of short branches (%)
PP	8.5 ± 0.3 b	3.2 ± 0.1 a	1.3 ± 0.2 b	10.5 ± 1.9 b	3.0 ± 1.0 b	27 ± 3 a	38 ± 21 b	33 ± 8 b	29 ± 14 a
RD	9.3 ± 0.6 ab	3.2 ± 0.1 a	1.3 ± 0.1 b	12.2 ± 0.7 b	3.7 ± 0.4 b	23 ± 3 ab	13 ± 4 c **↓**	72 ± 8 a ↑	14 ± 4 a
FH	8.7 ± 0.9 b	2.8 ± 0.1 b **↓**	1.4 ± 0.2 b	10.1 ± 1.0 b	3.3 ± 0.8 b	18 ± 2 b **↓**	20 ± 1 bc	62 ± 27 a ↑	17 ± 26 a
TN	10.8 ± 1.4 a ↑	3.1 ± 0.1 a	2.8 ± 0.4 a ↑	28.6 ± 5.0 a ↑	15.1 ± 3.7 a ↑	21 ± 5 ab	78 ± 12 a ↑	22 ± 12 b	0 ± 0 a

Different lowercase letters indicate significant differences at the level of 0.05. ↑ indicate the parameter increased, while ↓ indicate decreased compared with the PP.

**Table 2:** Branch structure parameters of Korla fragrant pear under different training systems.

Training systems	Total branch length (m)	Average branch length (cm)	Average diameter (cm)	Branch angle (°)	Average distance of branches (cm)	Total lateral branch number	Average lateral branch number
PP	19.4 ± 0.9 b	73 ± 14 b	2.1 ± 0.1 c	58 ± 6 b	9.9 ± 1.5 a	131 ± 6 b	5 ± 1 b
RD	16.4 ± 1.3 b	72 ± 3 b	2.6 ± 0.1 b ↑	72 ± 3 a ↑	11.2 ± 0.2 a	128 ± 18 b	6 ± 1 b
FH	13.0 ± 1.5 b	73 ± 9 b	2.7 ± 0.2 ab ↑	76 ± 3 a ↑	12.8 ± 1.6 a	114 ± 19 b	6 ± 1 b
TN	31.9 ± 7.0 a ↑	150 ± 18 a ↑	2.9 ± 0.1 a ↑	69 ± 4 a ↑	12.6 ± 2.6 a	208 ± 48 a ↑	10 ± 1 a ↑

Different lowercase letters indicate significant differences at the level of 0.05. ↑ indicate the parameter increased, while ↓ indicate decreased compared with the PP.

**Table 3:** Vertical distribution characteristics of branches of Korla fragrant pear under different training systems.

Training systems	Branch number			Average branch length (cm)			Average branch diameter (cm)			Average lateral branch number		
	Lower	Middle	Upper	Lower	Middle	Upper	Lower	Middle	Upper	Lower	Middle	Upper
PP	6 ± 3 a	9 ± 1 a	12 ± 2 a	83 ± 15 b	67 ± 20 b	73 ± 11 b	2.1 ± 0.1 b	1.9 ± 0.1 b	2.3 ± 0.2 b	7 ± 1 b	5 ± 1 b	4 ± 1 b
RD	6 ± 1 a	8 ± 3 a	9 ± 0 ab	69 ± 4 b	74 ± 8 b	74 ± 11 b	2.5 ± 0.2 b	2.5 ± 0.1 a ↑	2.7 ± 0.3 ab	6 ± 2 b	6 ± 1 ab	5 ± 2 b
FH	6 ± 1 a	7 ± 2 a	5 ± 2 c **↓**	88 ± 10 b	71 ± 19 b	62 ± 4 b	2.9 ± 0.2 a ↑	2.6 ± 0.4 a ↑	2.6 ± 0.5 b	7 ± 1 b	7 ± 2 ab	6 ± 0 b
TN	7 ± 1 a	8 ± 3 a	7 ± 2 b **↓**	159 ± 22 a ↑	129 ± 21 a ↑	164 ± 27 a ↑	2.9 ± 0.4 a ↑	2.5 ± 0.1 a ↑	3.3 ± 0.2 a ↑	12 ± 2 a ↑	8 ± 2 a ↑	9 ± 2 a ↑

Different lowercase letters indicate significant differences at the level of 0.05. ↑ indicate the parameter increased, while ↓ indicate decreased compared with the PP.

### 3.2 The influence of different training systems on the canopy light distribution of Korla fragrant pear

**Figure 3:**
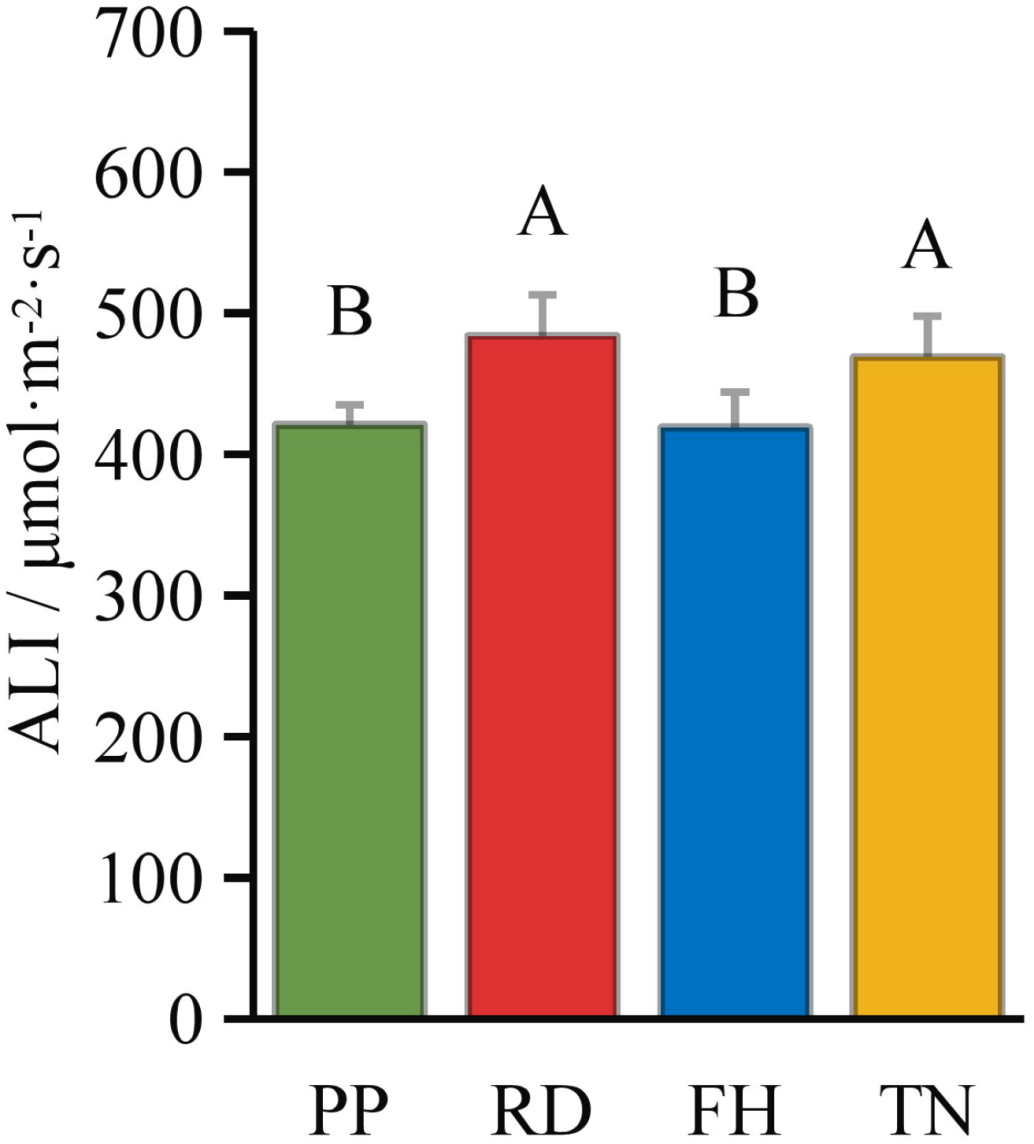
The ALI under different training systems.

**Figure 4:**
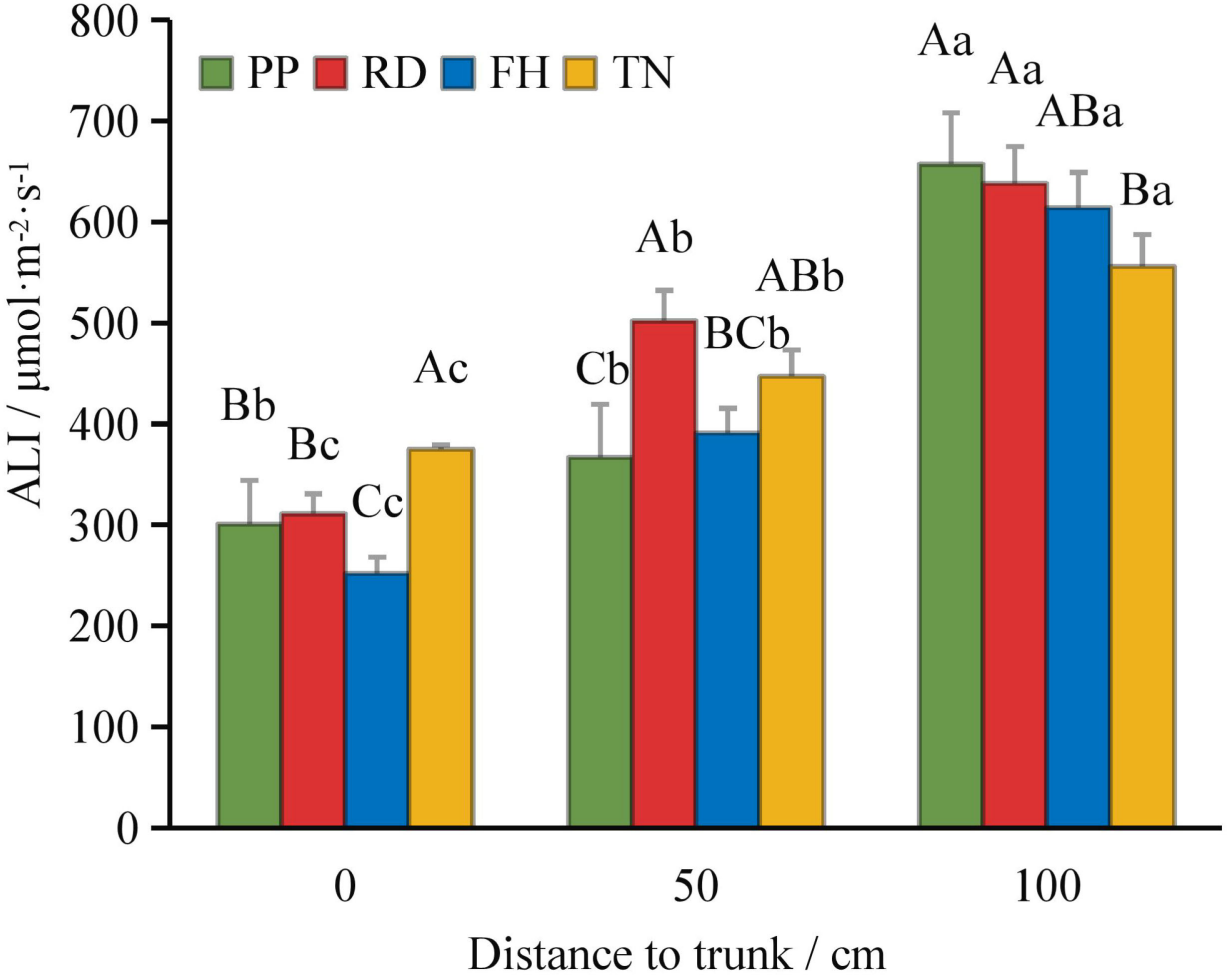
The ALI at different distance to trunk under different training systems.

**Figure 5:**
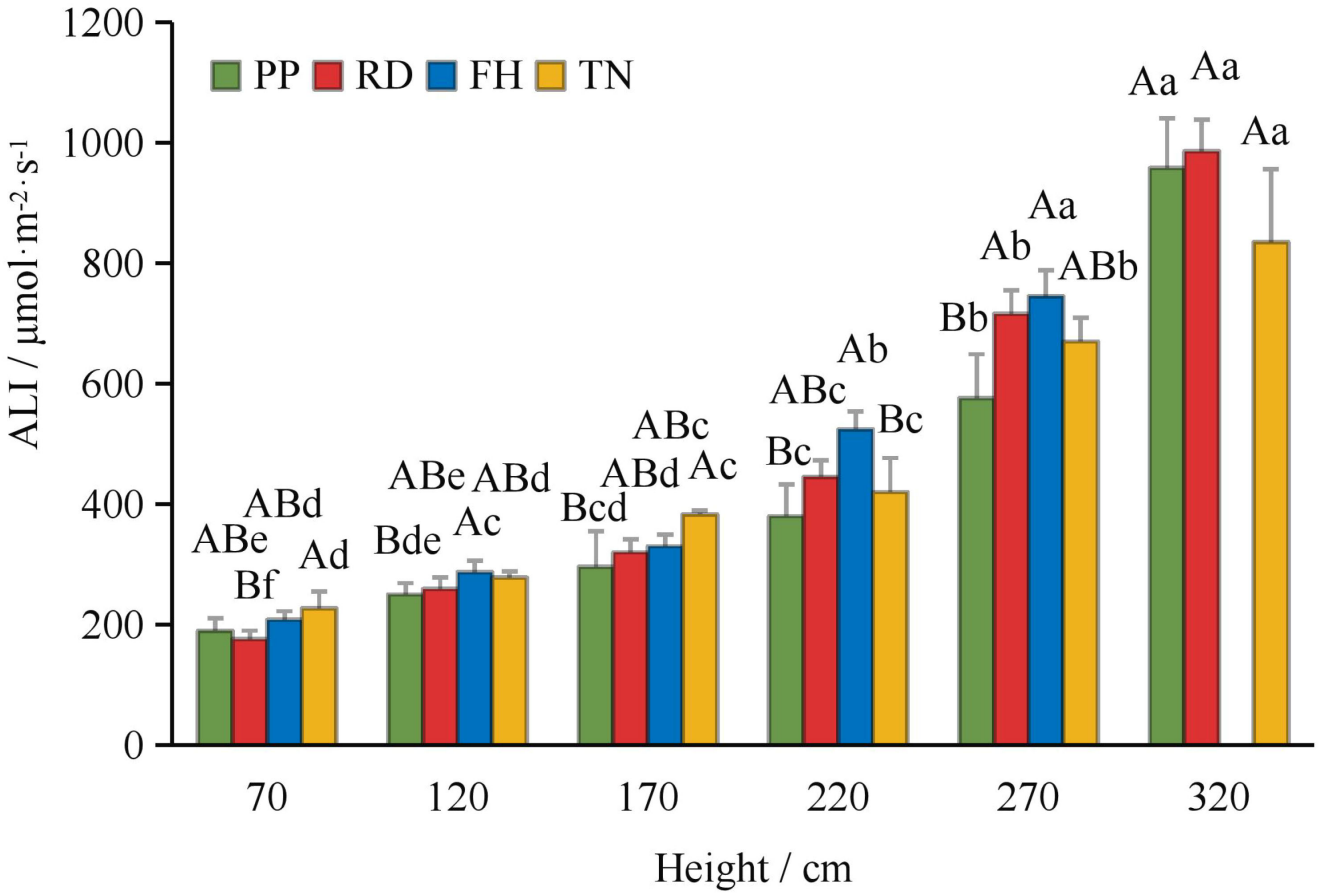
The ALI at different hight under different training systems.

**Figure 6:**
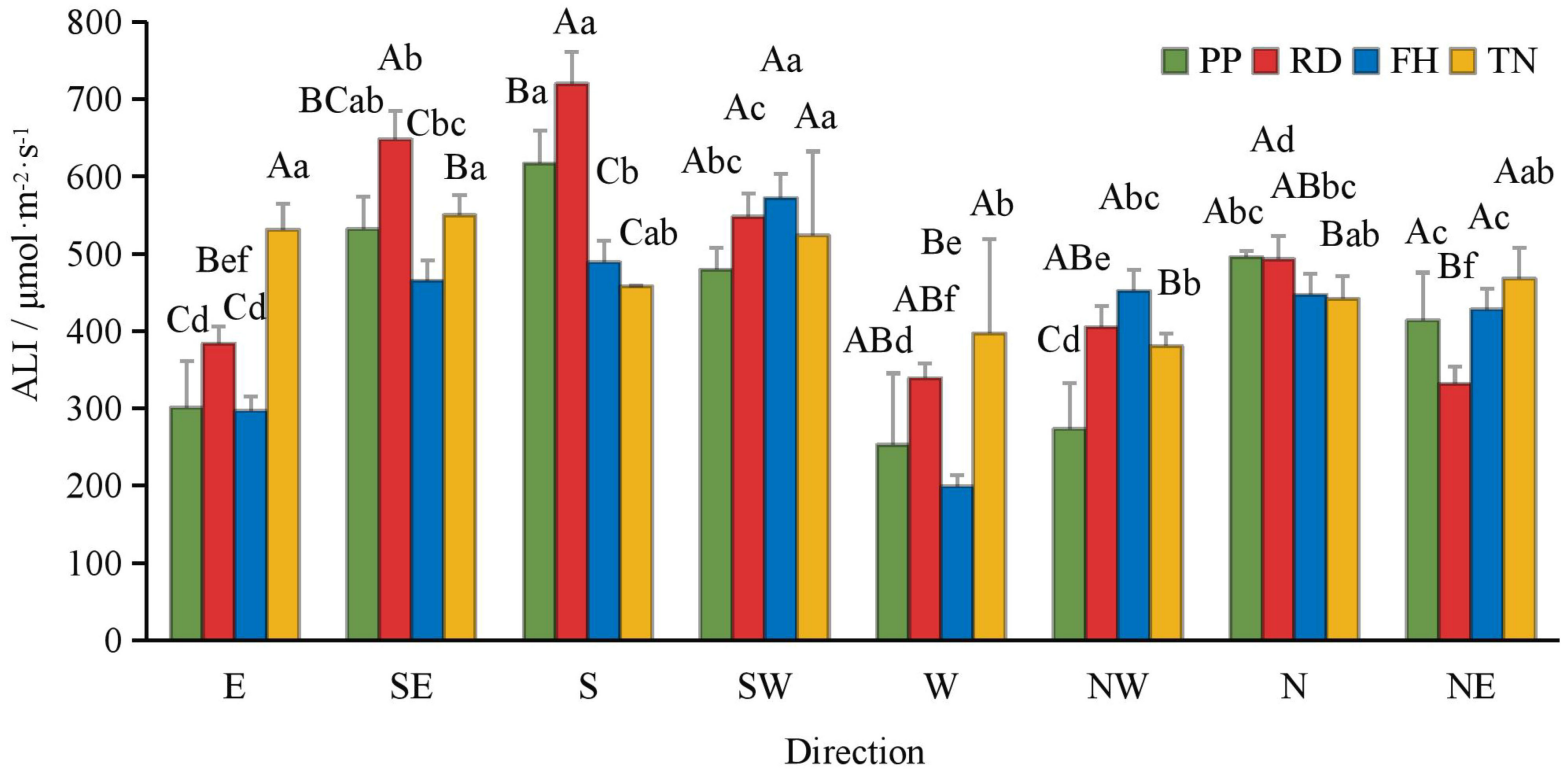
The ALI at different direction under different training systems. Different uppercase letters indicate that the PAR between different training systems was significantly different at the 0.05 level, and different lowercase letters indicate that the PAR between different parts was significantly different at the 0.05 level.

Compared to precision pruning (PP) (ALI = 421 μmol·m^-2^·s^-1^), both falling head (FH) and thinning (TN) significantly increased the canopy’s average light interception (ALI = 484 and 469 μmol·m^-2^·s^-1^, respectively; p < 0.05), while falling head (FH) resulted in an ALI of 419 μmol·m^-2^·s^-1^, showing no significant difference from precision pruning (PP) (**Figure 3**). Distance from the trunk: overall, ALI was significantly higher in the outer canopy than in the middle, and higher in the middle than in the inner canopy. Thinning (TN) significantly increased ALI in the inner canopy but decreased it in the outer canopy (p < 0.05) (**Figure 4**). Canopy height: ALI generally increased with height. Falling head (FH) significantly enhanced ALI at 120 cm, 220 cm, and 270 cm (p < 0.05) (**Figure 5**). Canopy aspect: overall, ALI was higher on the southern side than on the northern side, while the eastern and western sides exhibited lower ALI. Reduction (RD) significantly increased ALI on the eastern, southeastern, southern, and northwestern aspects but decreased it on the northeastern aspect. Thinning (TN) significantly increased ALI on the eastern and northwestern aspects but decreased it on the southern and northern aspects (p < 0.05) (**Figure 6**).

### 3.3 The influence of different training systems on the fruit spatial distribution of Korla fragrant pear

**Figure 7:**
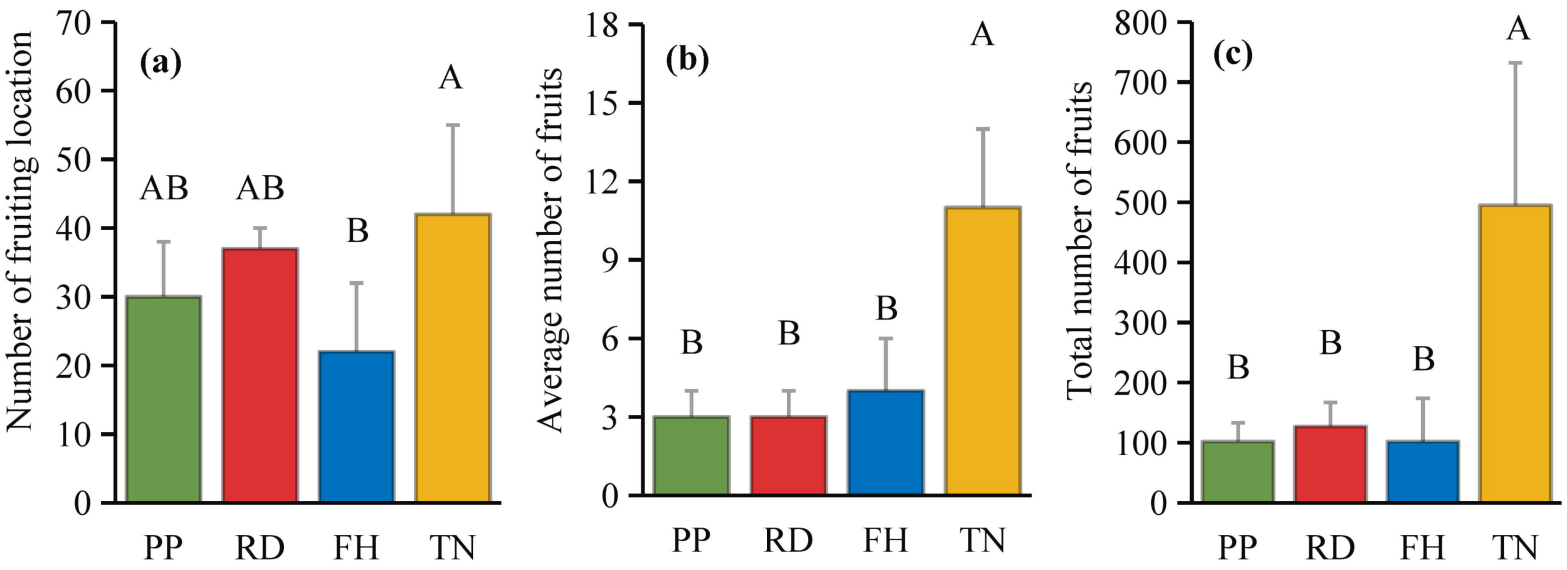
**(a–c)** the fruits number under different training systems.

**Figure 8:**
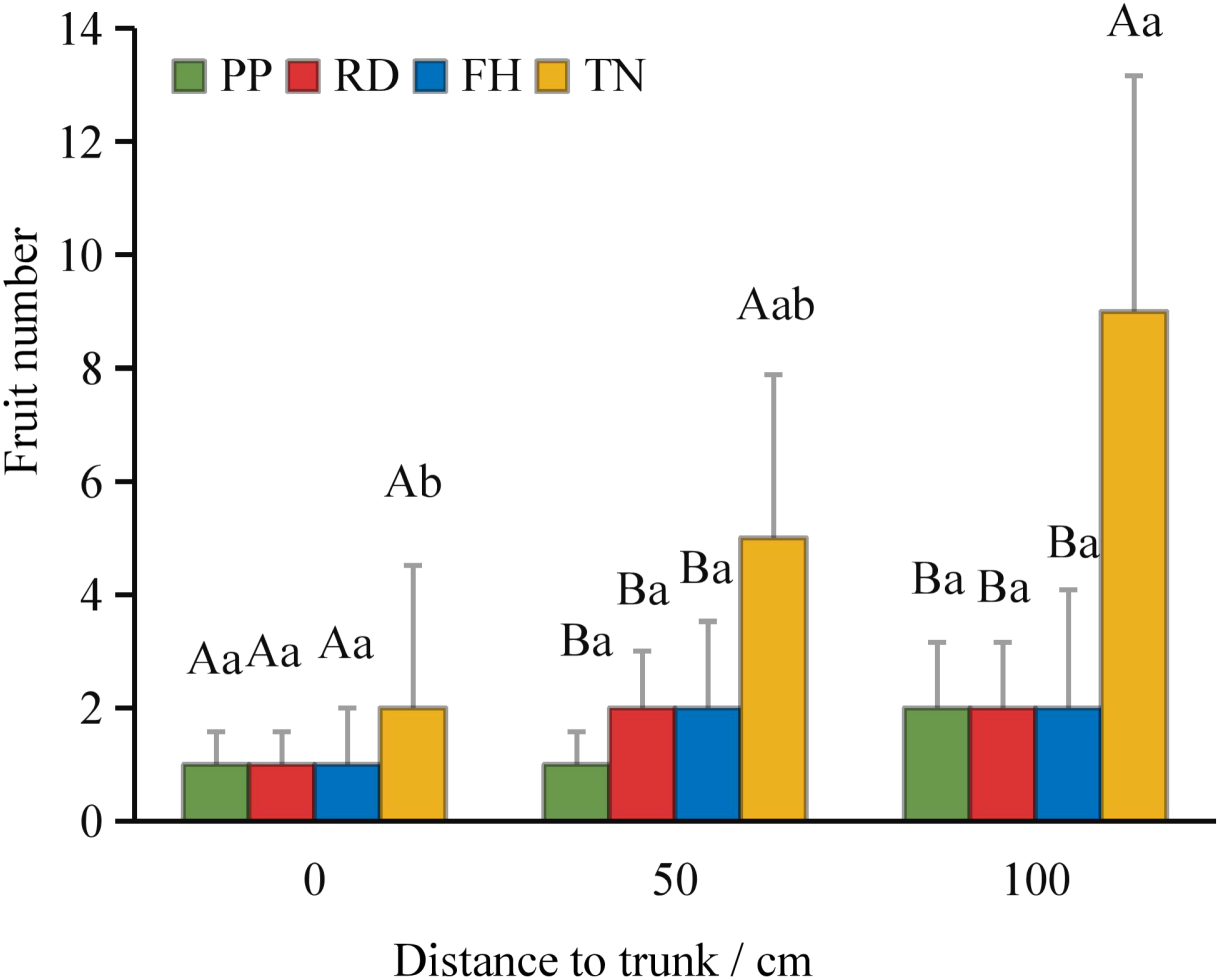
The fruit number at different distance to trunk under different training systems.

**Figure 9:**
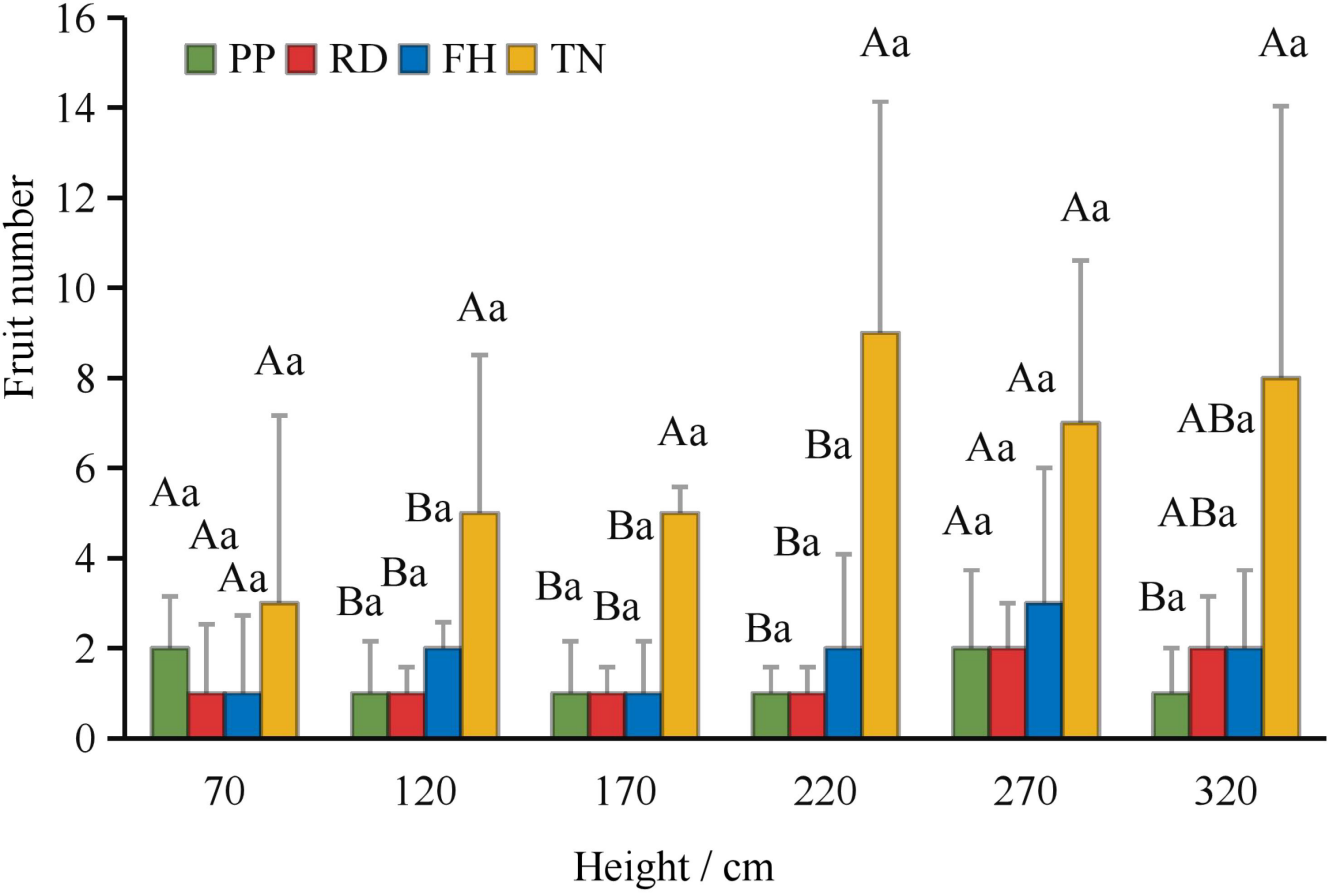
The fruit number at different hight under different training systems.

**Figure 10:**
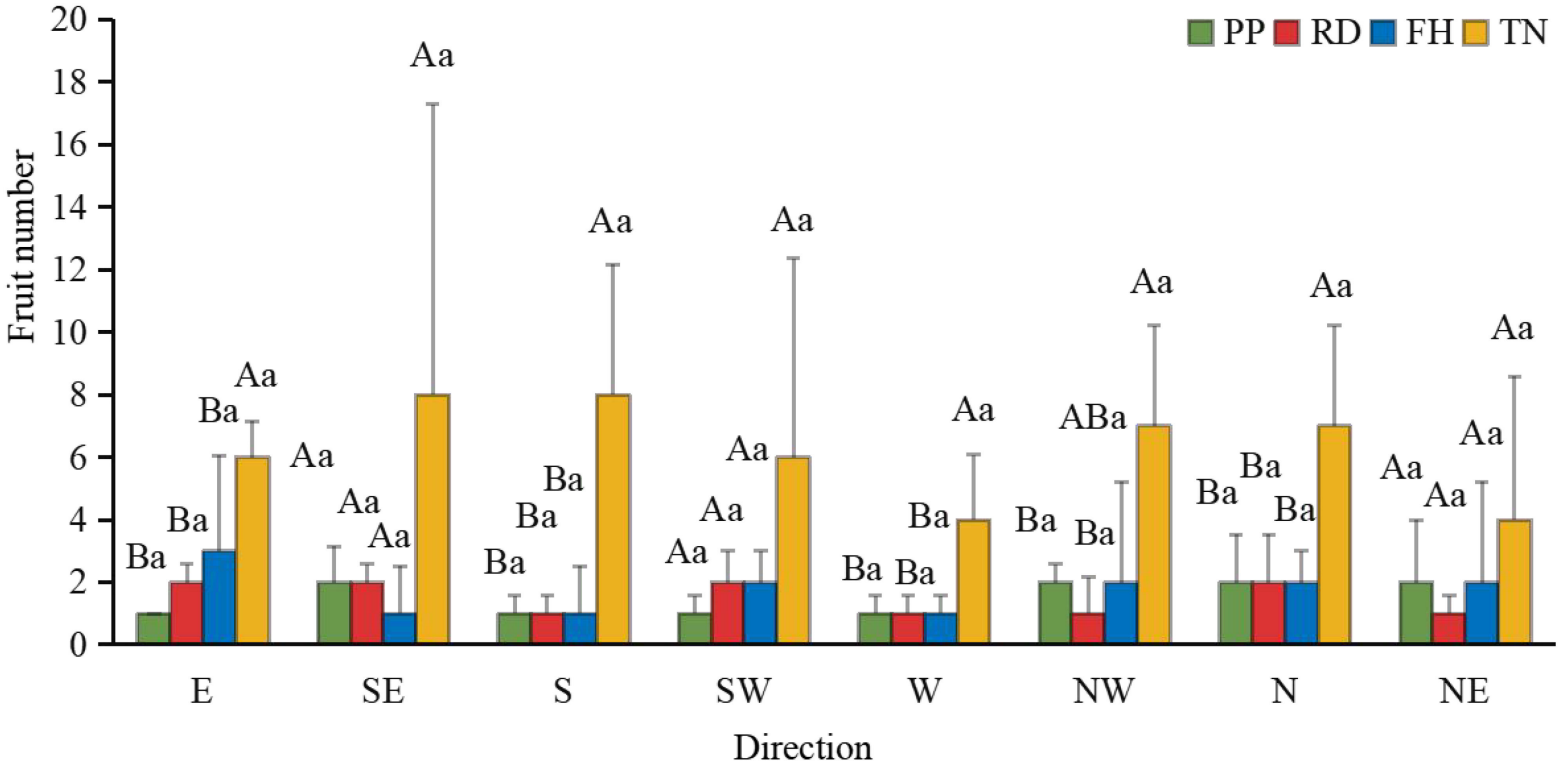
The fruit number at different direction under different training systems. Different uppercase letters indicate that the fruit number between different training systems was significantly different at the 0.05 level, and different lowercase letters indicate that the fruit number between different parts was significantly different at the 0.05 level.

Thinning (TN) significantly improved both average fruit number and total fruit number (p < 0.05) (**Figures 7a–c**). Distance from the trunk: Thinning (TN) significantly enhanced fruit numbers at 50 cm and 100 cm distances from the trunk (p < 0.05), with the majority of fruits concentrated in the middle and outer canopy regions (**Figure 8**). Canopy height: vertical distribution significant increases in fruit numbers were observed at multiple canopy heights after thinning: 120 cm, 170 cm, 220 cm, 320 cm (all p < 0.05) (**Figure 9**). (3) Canopy aspect: Thinning (TN) significantly increased fruit numbers across multiple aspects: Eastern, Southern, Western, Northwestern, Northern (p < 0.05) (**Figure 10**).

### 3.4 The influence of different training systems on the photosynthesis and consistently high yield traits

**Figure 11:**
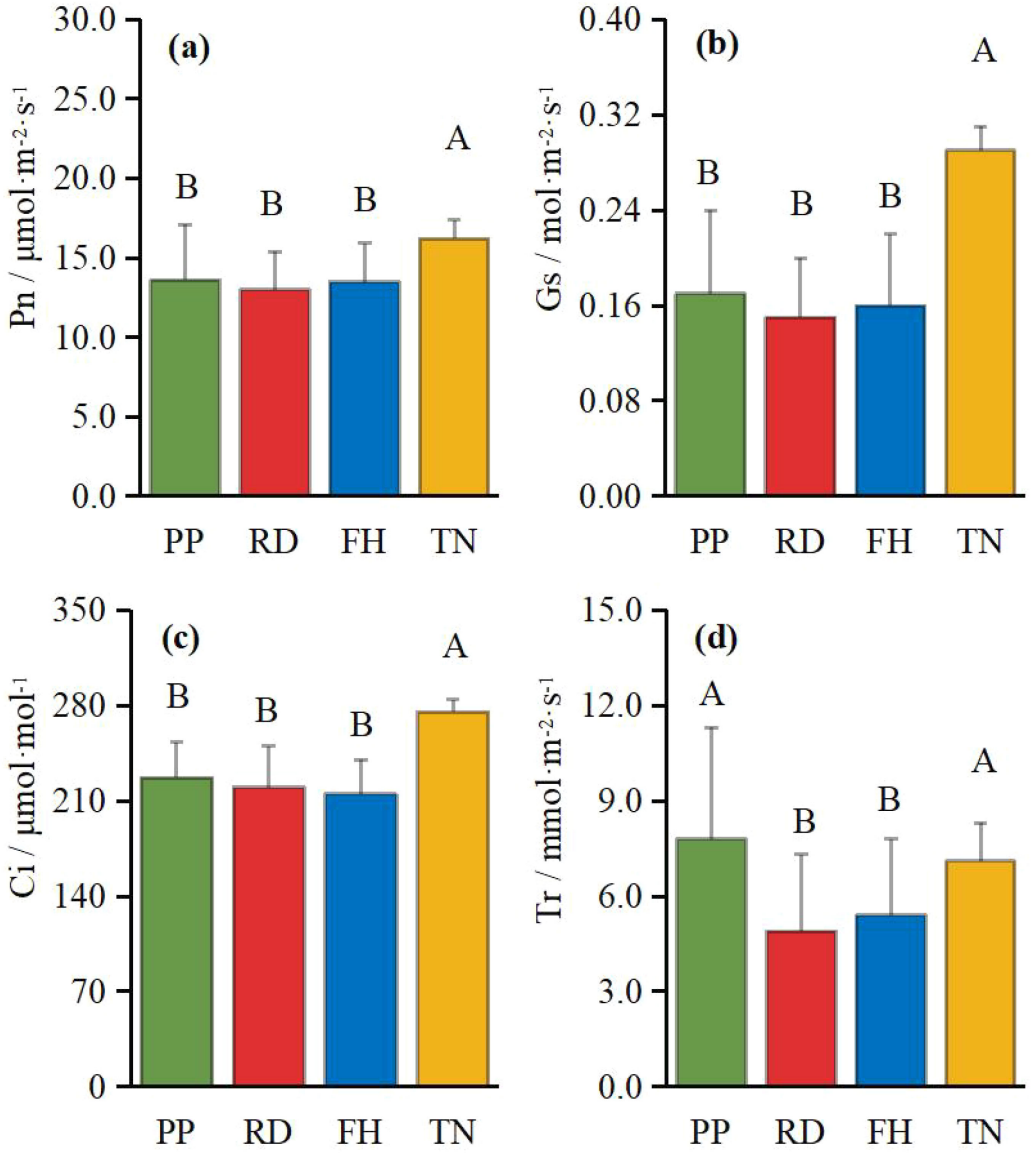
**(a–d)** the photosynthesis under different training systems.

**Figure 12:**
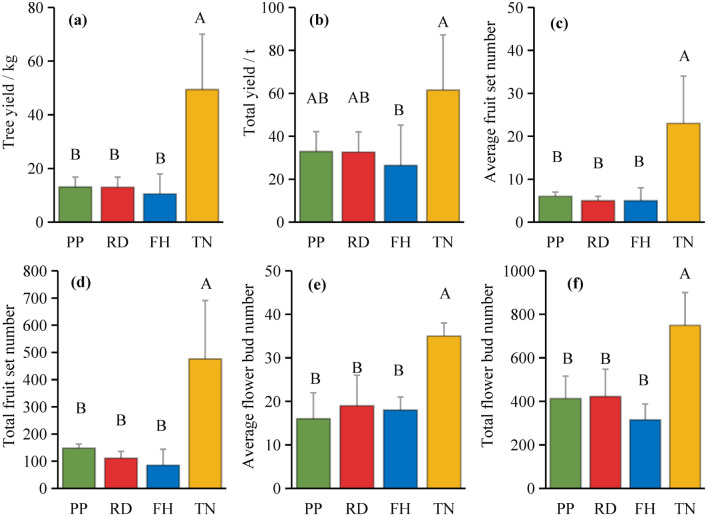
**(a–f)** the consistently high yield traits under different training systems.

**Figure 13:**
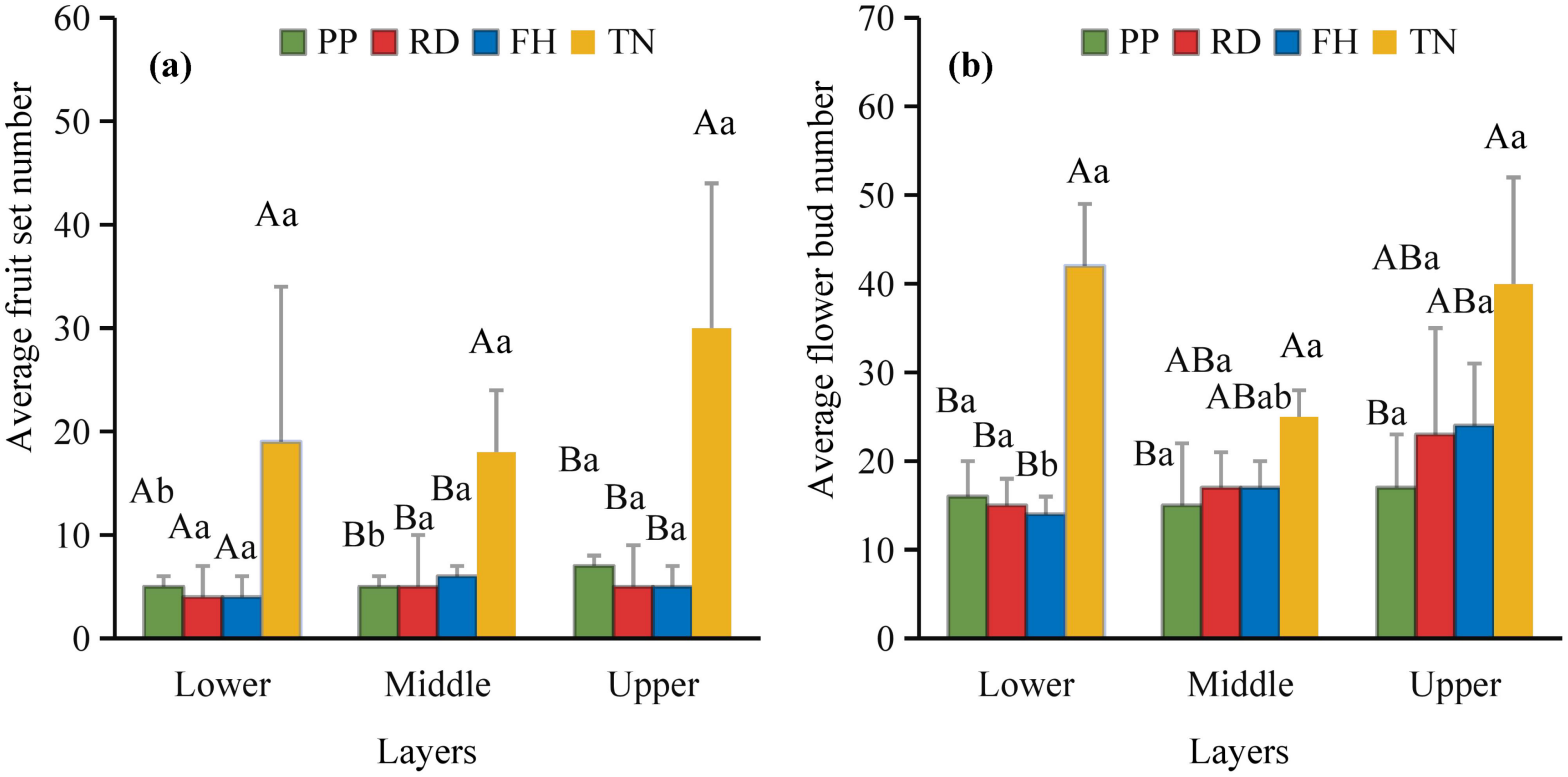
**(a, b)** the vertical distribution characteristics of fruit and flower buds in the second year.

Thinning (TN), compared to precision pruning (PP), the canopy expansion space increased and the leaf light environment was optimized. Photosynthetic rate significantly increased by 20%, stomatal conductance rose by 70%, and intercellular CO_2_ concentration elevated by 20%. Although transpiration rate remained relatively stable, it was significantly higher than both reduction (RD) and falling head (FH). In summary, thinning enhanced leaf carbon assimilation capacity, providing sufficient organic compounds for carbon allocation (**Figures 11a–d**). Thinning (TN) significantly enhanced most yield parameters compared to precision pruning (PP) (p < 0.05). The treatment resulted in: 3.8-fold increase in yield per tree, 1.9-fold increase in total yield, 3.8-fold increase in average fruit set, 3.2-fold increase in total fruit number, 2.2-fold increase in average flower bud number, 1.8-fold increase in total flower bud number (**Figures 12a–f**). Vertical distribution patterns: Thinning (TN) significantly increased fruit numbers in the middle and upper canopy layers (p < 0.05), middle layer: 3.6-fold increased, upper layer: 4.3-fold increased, these layers became the primary fruit-bearing zones. All canopy layers showed significant increase in flower bud numbers (p < 0.05), lower layer: 2.6-fold increased, middle layer: 1.7-fold increased, upper layer: 2.4-fold increased (**Figures 13a, b**).

### 3.5 Relationship between canopy structure and fruit yield

**Figure 14:**
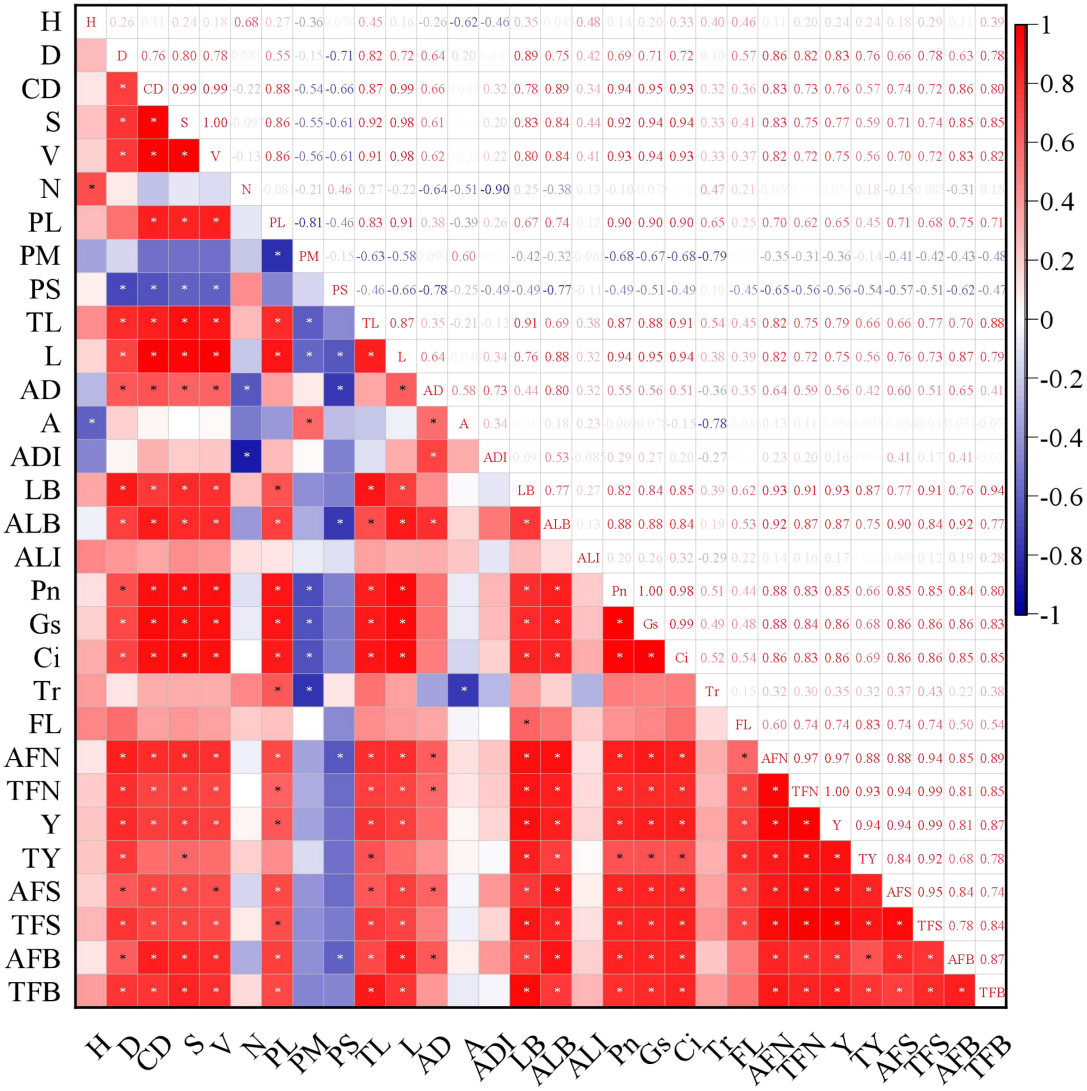
The correlation between canopy structure and light interception as well as yield. * represents a significant correlation at the level of 0.05 (bilateral). H, Height; D, Diameter; CD, Canopy diameter; S, Canopy surface area; V, Canopy volume; N, Branch number; PL, Proportion of long branches; PM, Proportion of middle branches; PS, Proportion of short branches; TL, Total length of branches; L, Average branch length; AD, Average diameter; A, Branch angle; ADI, Average distance of branches; LB, Total lateral branch number; ALB, Average lateral branch number; ALI, Average light interception; Pn, Net photosynthetic rate; Gs, Stomatal conductance; Ci, Intercellular CO_2_ concentration; Tr, Transpiration rate; FL, Fruit location; AFN, Average fruit number; TFN, Total fruit number; Y, Yeild; TY, Total yeild; AFS, Average fruit set; TFS, Total fruit set; AFB, Average flower bud number; TFB, Total flower bud number.

Two canopy structural parameters showed negative correlations with average fruit number: proportion of middle branches (non-significant), proportion of short branches (significant, p < 0.05). Ten canopy structural parameters exhibited significant positive correlations (p < 0.05) with average fruit number: diameter, canopy diameter, canopy surface area, canopy volume, proportion of long branches, total length of branches, average branch length, average diameter (branch), total lateral branch number, average lateral branch number (**Figure 14**). Notable findings, three structural parameters showed significant positive correlations with total yield: tree height, canopy surface area, total branch length. Photosynthesis was positively correlated with fruit number, yield and flower bud number. Except for transpiration rate (Tr), all correlations reached a significant level (p < 0.05). The enhancement of photosynthesis improves carbon assimilation efficiency, thereby providing ample substrates for carbon allocation and ensuring both current-year fruit yield and floral bud formation. Thinning (TN) treatment demonstrated the highest light interception capacity and photosynthetic performance, consequently achieving the maximum yield. These results indicate that optimizing canopy growth space and structure not only improves the light environment within the canopy but also enhances the photosynthetic functionality of leaves.

### 3.6 Relationship between canopy light distribution and fruit spatial distribution

**Figure 15:**
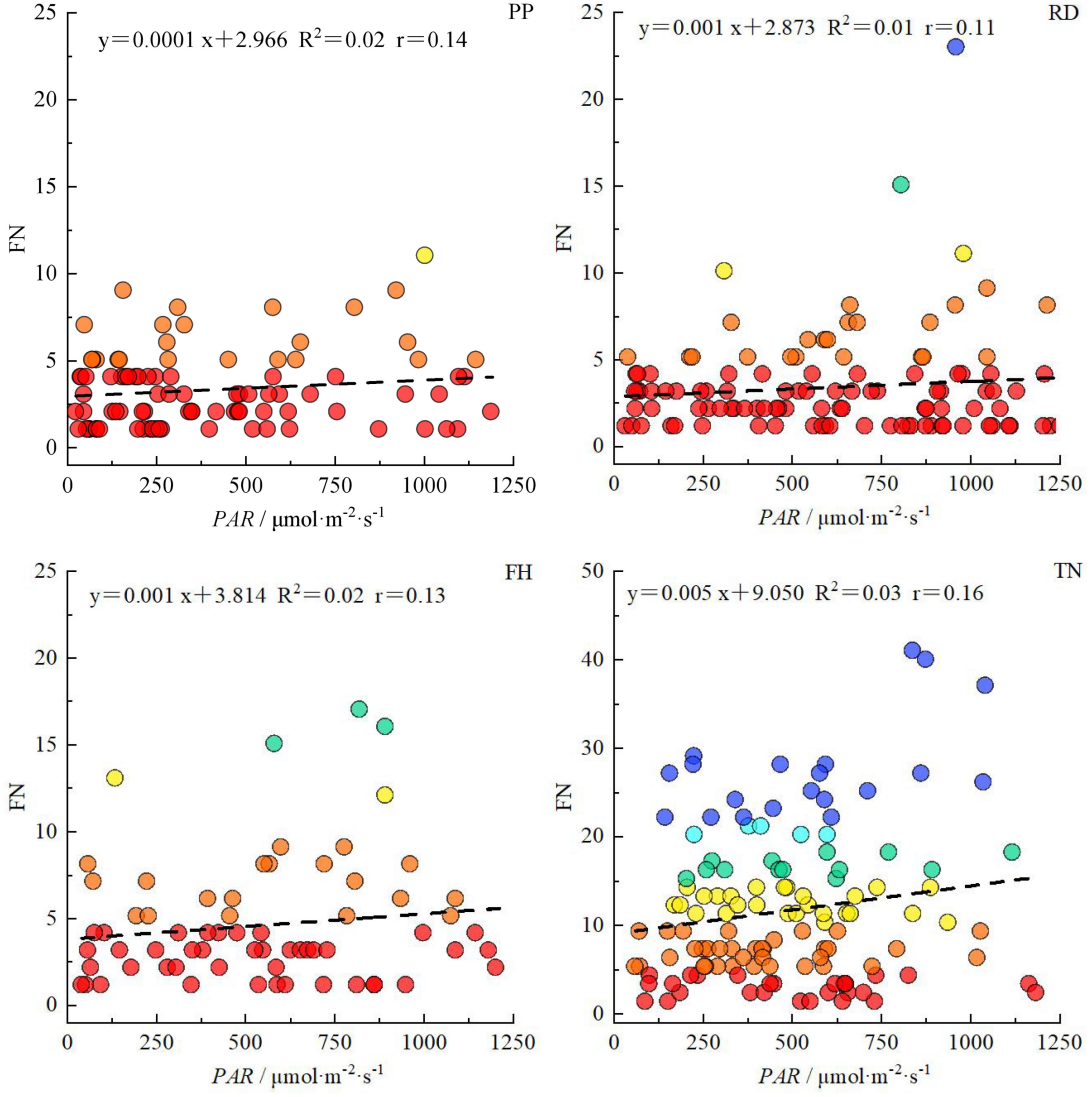
The correlation between *PAR* and fruit number in canopy under different training systems. R^2^ represents the determination coefficient of the equation, and r represents the correlation coefficient between PAR and fruit number.

**Figure 16:**
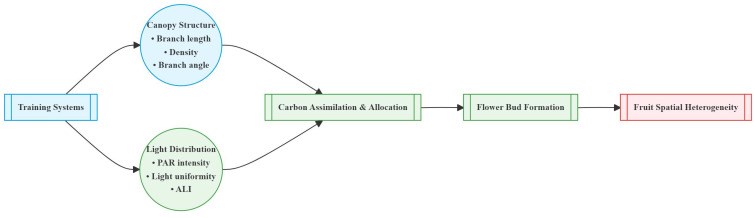
Conceptual model of canopy structure and light distribution-driven fruit spatial heterogeneity.

Overall, the correlation between light distribution and fruit distribution was weaker in small canopies but stronger in large canopies. Reduction (RD) and Falling head (FH) reduced the correlation between PAR and fruit number, while Thinning (TN) enhanced it (**Figure 15**). The horizontal correlation between PAR and fruit number decreased with canopy narrowing but increased with canopy expansion (**Table 4**). For small canopies, canopy structure was the primary factor affected fruit spatial distribution, whereas for large canopies, light distribution was the primary factor affected fruit spatial distribution (**Figures 15**, **16**). In Precision Pruning (PP) canopies, PAR showed a strong correlation with fruit number at different distances from the trunk (r = 0.63). After Reduction (RD), the correlation between PAR and fruit number weakened at different distances from the trunk (r = 0.09), but strengthened at different heights (r = 0.38). Falling head (FH) showed similar trends to Reduction (RD). After Thinning (TN), the correlation between PAR and fruit number increased both at different distances from the trunk (r=0.71) and at different heights (r=0.21).

**Table 4:** The correlation model between *PAR* and fruit number in different canopy parts.

Training systems	Parts	Slope a	Intercept b	R^2^	r
PP	Distance to trunk	0.003	-0.139	0.39	0.63
	Height	-0.0003	1.557	0.004	-0.07
	Direction	-0.0002	1.505	0.0008	-0.03
RD	Distance to trunk	0.0005	1.413	0.008	0.09
	Height	0.001	1.036	0.14	0.38
	Direction	-0.0007	1.371	0.01	0.11
FH	Distance to trunk	0.003	0.581	0.08	0.28
	Height	0.002	1.001	0.06	0.24
	Direction	-0.0003	1.976	0.0004	-0.02
TN	Distance to trunk	0.036	-11.019	0.51	0.71
	Height	0.004	4.615	0.04	0.21
	Direction	-0.007	9.210	0.01	-0.12

## 4 Discussion

### 4.1 Variation in canopy structure of Korla fragrant pear trees

Different training systems created tree shapes with different canopy structures. Reduction (RD) narrowed the canopy, Falling head (FH) reduced its height, and Thinning (TN) expands its width. Notably, Thinning (TN) increased canopy volume by fourfold, demonstrated the most significant structural modification. These findings indicated that training systems, particularly those regulating planting density, profoundly influence canopy development. When density remains constant, only minor parameter adjustments occur, resulting in limited changes in canopy structure. Thus, the regulatory effect of population structure outweighs that of individual tree structure. Thinning is an effective practice that promotes flower bud differentiation, enhances the formation of long and middle fruiting branches, and significantly improves individual tree yield (Tian, 2007; Zhang, 2015). This study observed a substantial increase in long branches and fruiting sites after Thinning (TN), led to higher productivity. Among various canopy parameters, branch type composition and length were key factors derived structural differences. Maintaining appropriate branch length in Korla fragrant pear is crucial for yield formation. Necessitating optimized planting systems, such as wider row and plant spacing. Additionally, canopy structure evolves with tree age. Studies on high-spindle apple trees aged 3, 6, 9, and 11 years revealed that trunk girth, crown diameter, canopy volume, and leaf density increased over time (Liang et al., 2011). Similarly, this study found that the proportion of long and middle branches in Korla fragrant pear increased with age, expanding the canopy. However, to mitigate shading, some branches were removed, reducing overall branch density. Considering interannual canopy dynamics is essential for optimizing yield. For instance, high-density apple orchards in early fruiting stages exhibit higher yields due to greater shoot numbers, but this advantage diminishes with age (Li et al., 2020). A similar trend occurs in fragrant pear—once the canopy stabilizes, yield declines, likely due to increased low-light zones (relative light intensity <30%) and altered branch distribution (Shang et al., 2010). Therefore, canopy structure should be continuously optimized through height control, branch thinning, and moderate pruning (Zhao et al., 2011; Zhu, 2013; Zhang, 2017). Here, we propose that while reduction reduces canopy size, facilitating mechanized operations, it may lower yields. To enhance productivity, reducing the intensity of reduction while appropriately extending branches and enlarging the canopy is recommended. In apple cultivation in Southern Xinjiang, the wide-row, dense-planting system shares similarities with the labor-saving Korla fragrant pear cultivation model. Both adopt a spindle-shaped tree structure, consisting of a central leader with multiple fruiting branches. Their pruning techniques are also comparable, featuring monopodial extension combined with training methods such as branch opening, bending, heading-back, thinning, and topping (Yan et al., 2022). However, as their canopies expand, they similarly face issues of overcrowding. Therefore, the findings and canopy management strategies presented in this study also hold significant implications for optimizing modern apple production systems.

### 4.2 Canopy light distribution patterns in Korla fragrant pear trees

Implications for light availability is the most critical factor in tree management, as canopy structure directly influences light interception and distribution, thereby shaping the micro-environment within the canopy (Gao, 2020; Zhang, 2020). In Korla fragrant pear, light distribution exhibited distinct horizontal, vertical, and aspect patterns. Generally, the middle and outer canopy received better illumination, with higher average light interception (ALI) on the south side due to row orientation, while the east and west sides exhibit lower ALI, primarily influenced by plant spacing. Different training systems modulated light distribution differently: Reduction (RD) and Thinning (TN) improved light penetration in specific aspects, though their effects vary spatially. Falling head (FH) enhanced light availability in the middle and lower canopy layers. Precision pruning (PP) leads to an 18% reduction in ALI (compared to young trees with a high proportion of short branches, ALI = 514 μmol·m^-^²·s^-^¹) (Yan et al., 2024), likely due to increased long branch density, which impedes light penetration. A similar trend was observed in high-spindle apple trees, where sky view factor decreases with age (Liang et al., 2011). Reduction (RD) reduced inter-tree shading, minimized branch overlap within a narrow 1 m spacing while restricted row-side expansion, thereby improving light penetration into typically shaded areas (east, west, north, and lower canopy). This increased ALI at 50 cm, aligning with findings that thinning the canopy enhances light transmission in olive trees (Trentacoste et al., 2018; Rallo et al., 2024). Falling head (FH) lowered tree height, facilitated deeper light penetration and more uniform ALI distribution in the middle and lower canopy, consistent with studies advocating height control in slender spindle apple systems (Han et al., 2001; Yoon and Bhusal, 2016; Bhusal et al., 2019). Thinning (TN), despite increased long branch density, expanded plant spacing and reduced branch number, allowed more light to enter from inter-tree gaps (east and west sides). This significantly improved ALI at 0 cm and 50 cm, counteracted the decline in inner-canopy illumination as the canopy expands (Wagenmakers and Callesen, 2015). Optimal relative light intensity (RLI) for high-quality yields in open-center trained fragrant pear ranges between 46–73%, with 72.48% being ideal (Cheng, 2013; Liu et al., 2014). Our study revealed that while light intensity showed a positive correlation with yield in Precision Pruning (PP), Reduction (RD), and Falling head (FH) trees, the relationship was relatively weak. This may be attributed to the underdeveloped canopy structure in these treatments, where yield remained primarily determined by architectural parameters rather than light availability. These findings align with previous observations by Lu chao (Lu et al., 2011), who similarly reported no significant correlation (r = 0.5) between fruit number and light intensity in comparable training systems. Nevertheless, most research supports the benefits of improved light exposure. For instance, well-illuminated apple canopies exhibit higher fruiting efficiency and flower bud formation (Zhang et al., 2010). Similarly, Thinning (TN) in this study enhanced light conditions and fruit set, reinforcing the importance of light management. In apple cultivation in southern Xinjiang, a similar pattern of canopy light distribution has been observed: as the canopy expands, the heterogeneity of light distribution increases (Yan et al., 2022). The method proposed in this study—improving canopy light conditions—also provides valuable insights for modifying in closely planted apple orchards with wide row spacing.

### 4.3 Spatial distribution characteristics of fruits in Korla fragrant pear trees

Canopy structure influences photosynthetic efficiency and ultimately yield by regulating light distribution within the tree crown (Zhang, 2020). Our results demonstrate that thinned canopies exhibited significantly higher fruit numbers in middle and upper layers, as well as peripheral regions, corresponding with areas of optimal light penetration. This aligns with previous findings showing superior fruit production in well-illuminated upper and outer canopy zones of fragrant pear (Cheng, 2013; Liu et al., 2014) and apple trees (Ma, 2012). However, some studies report discordance between light distribution patterns and yield allocation (Farina et al., 2005; Geng et al., 2009). This study found that, small canopies showed weak light-yield correlations, large canopies exhibited strong spatial correspondence between light availability and fruit distribution. At different aspects, southern aspects produced more fruit than northern aspects, eastern/western aspects showed reduced yields due to lower light availability. This finding aligns with the conclusion that branches with higher light availability at different orientations tend to bear more fruits (Chaves et al., 2011). Among all treatments, the Thinning (TN) canopy exhibited a higher fruit number in all orientations, with significantly more fruits on the eastern, southern, western, and northern aspects compared to other treatments. This is likely associated with improved light conditions on the eastern and western aspects of the canopy after Thinning (TN). Previous studies have demonstrated that the number of branches significantly affects apple yield and its spatial distribution (Zhang et al., 2014). However, in this study, the correlation between branch number and fruit count was weak, likely due to the unique characteristics of the Thinning canopy, which exhibited fewer branches but a higher fruit load. Previous studies have also shown that tall spindle apple trees with a higher number of lateral branches exhibit greater early yield compared to slender spindle, free spindle, and modified spindle systems (Dong et al., 2013). In this study, the Thinning (TN) canopy had the highest number of lateral branches, which was significantly positively correlated with fruit number, consistent with these findings. This study further revealed a negative correlation between the proportion of middle and short branches and fruit number. However, previous research demonstrated that short branches significantly enhance light interception efficiency. These findings suggest that while short branches improve canopy light penetration, their limited bearing capacity may restrict yield potential. Thus, optimizing the balance between light capture and productivity requires careful adjustment of long-, middle-, and short-branches ratios to achieve optimal orchard performance (Yan et al., 2024). Moreover, canopy structure is dynamic, and the key parameters determining yield vary with tree age, leading to differences in productivity (Chen, 2019; Liang et al., 2011). Future studies should incorporate long-term observations and expand the range of canopy structure types to establish yield prediction models. This will help identify key branch parameters and canopy structural traits associated with high productivity, thereby improving orchard management practices. Economic Evaluation: Precision pruning (PP), Reduction (RD), Falling head (FH) and Thinning (TH) achieved yields of 32.9 t/ha, 32.6 t/ha, 26.4 t/ha and 61.5 t/ha, respectively. Premium fruit rate (single fruit weight > 100 g): 90%, 80%, 80% and 70%, respectively. Assuming a unit price of $0.5/kg, gross output value: $15k/ha, $13k/ha, $11k/ha and $22k/ha, respectively. Production costs: $0.9k/ha, $0.75k/ha, $0.75k/ha, and $1.05k/ha, respectively. Net profit: $14k/ha, $12k/ha, $10k/ha, and $20k/ha, respectively. Compared to Reduction (RD), Thinning (TN) increased profit by $8k/ha (a 70% growth rate), demonstrating significant economic benefits. Although Thinning (TN) slightly raised labor costs due to reduced planting density, overall management costs did not increase substantially. Notably, Thinning (TN) led to a lower premium fruit rate, supporting our hypothesis that large-canopy systems require optimized light distribution and fruit spatial arrangement to enhance quality. These findings validate the proposed canopy management strategies for improving both yield and fruit grade.

### 4.4 Research prospects

This study was conducted at the individual tree scale, with detailed characterization of canopy structure, light distribution, and fruit spatial distribution based on three standard trees per treatment. The findings are generalizable to fruit tree individuals with similar canopy structures. However, compared to an orchard population comprising thousands of trees, three standard trees—even if carefully selected—remain a limited sample size. Future research should refine the methodology by adopting advanced measurement technologies to improve efficiency, expand the sampling scope, and conduct more studies at the population scale to better represent orchard-wide conditions. Korla fragrant pear has two main production regions: the Korla region and the Aksu region. This study was conducted in the Korla region, and whether the conclusions apply to the Aksu region requires further validation due to climatic and edaphic differences between the two areas. According to surveys, both regions experience a temperate continental climate. However, compared to Korla, Aksu has slightly higher annual precipitation (approximately 70–100 mm), milder winter temperatures, and a higher accumulated temperature (≥10°C), which may lead to earlier fruit ripening. In terms of soil, some areas in Aksu have higher clay content and poorer permeability compared to the sandy loam soils of Korla, potentially resulting in shallower root distribution and differences in canopy development and yield. Overall, from an aboveground perspective, the environmental conditions are similar, and the canopy structure and light distribution regulation methods proposed in this study may serve as a reference. However, attention should be paid to belowground differences, as soil texture variations may affect root development and, consequently, canopy growth. Therefore, cross-regional studies on canopy regulation are warranted.

## 5 Conclusion

(1) Under different training systems, canopy structures exhibited significant variations. Both Reduction (RD) and Falling Head (FH) effectively controlled canopy diameter, significantly reducing the proportion of long branches while increasing medium branch proportion. In contrast, Thinning (TN) markedly increased long branch proportion, total branch length, and average branch length, thereby expanding canopy diameter, surface area, and volume. (2) Under different canopy structures, canopy light distribution exhibited significant variations. Reducing canopy spread and increasing planting spacing improved light distribution across different aspects, while lowering canopy height enhanced light availability in middle and lower layers. Light interception varied markedly across canopy zones, with lower layers, inner canopy, and inter-tree spaces constituting low-light regions. (3) Smaller canopies exhibited reduced light interception area and photosynthetic capacity, leading to lower yields but more uniform fruit spatial distribution; whereas larger canopies demonstrated enhanced light capture and photosynthetic activity, resulting in higher productivity at the expense of decreased fruit distribution uniformity. (4) The spatial distribution of fruits in small canopies is primarily regulated by canopy structure, while in large canopies, it is mainly influenced by light distribution. Small canopies need to increase canopy surface area and total branch length to enhance fruiting sites and boost yield, whereas large canopies require optimized light distribution to improve the uniformity of fruit spatial distribution. (5) Thinning significantly improved consistently high yield traits by increasing the proportion of long branches and the number of lateral branches, thereby promoting flower bud differentiation.

## Data Availability

The original contributions presented in the study are included in the article/supplementary material. Further inquiries can be directed to the corresponding author.
